# Health Care Utilization in Diverse Older Adults in the Deep South and the Rest of the United States

**DOI:** 10.1093/geroni/igab046.380

**Published:** 2021-12-17

**Authors:** Maria Pisu, Roy Martin, Liang Shan, Giovanna Pilonieta, Richard Kennedy, Gabriela Oates, David Geldmacher

**Affiliations:** 1 University of Alabama at Birmingham, Birmingham, Alabama, United States; 2 University of Alabama at Birmingham, BIrmingham, Alabama, United States; 3 University of Alabama At Birmingham, Birmingham, Alabama, United States; 4 University of Alabama at Birmingham, University of Alabama at Birmingham, Alabama, United States

## Abstract

We examined racial/ethnic (R/E) differences in health care utilization among older adults with Alzheimer’s disease and related dementia (ADRD) from US Deep South [DS] and non-DS, and individual or context-level factors that affect this utilization. Data were 2013-2015 claims for Medicare beneficiaries with ADRD; county-level data were used to define context-level covariates; adjusted analyses were conducted separately for DS and non-DS. Across R/E groups, 33%-43% in DS, 43%-50% in non-DS used ADRD specialists; 47%-55% in DS, 41%-48% in non-DS used ADRD drugs; 42.9%-53.4% in DS, 42%-51.8% in non-DS had hospitalizations in a one-year follow-up. R/E differences were not significant, with few exceptions. Comorbidities, poverty, and medical resources availability were associated with specialist use and hospitalizations; comorbidities and specialist use were associated with drug use. In non-DS only, other individual, context-level covariates were associated with health care outcomes. Research should further examine determinants of health care utilization in these populations.

